# A postgraduate training programme in child and adolescent mental health in a lower-middle-income country: a partnership model from Nigeria

**DOI:** 10.1192/bji.2023.27

**Published:** 2024-02

**Authors:** Olayinka Omigbodun, Cornelius Ani

**Affiliations:** 1 MPH, FWACP, MD Professor of Psychiatry and Founding Director, Centre for Child and Adolescent Mental Health, University of Ibadan, Ibadan, Nigeria. Email: olayinka.omigbodun@gmail.com; 2 FRCPysch, MD, Consultant Child and Adolescent Psychiatrist and Honorary Clinical Senior Lecturer, Division of Psychiatry, Imperial College London, London, UK

**Keywords:** Nigeria, low- and middle-income countries, child and adolescent mental health, curriculum, postgraduate teaching

## Abstract

This paper describes a postgraduate training programme in child and adolescent mental health (CAMH) in Nigeria. It explains the background, curriculum development, teaching, evaluation and outcomes. By its 10th year the programme had trained 166 CAMH professionals from 14 African countries. Many of the graduates are running clinical CAMH services in their countries, mostly pioneered by them. They are also conducting CAMH training, including as faculty on the programme, and some are in international CAMH leadership roles. Key success elements of the programme that can be replicated in other low- and middle-income countries include international partnership, adopting a train-the-trainer approach, using a curriculum that covers clinical aspects of CAMH while also developing leadership and research skills, use of free-access training resources, and access to seed funding.

Nigeria has a young population, with 50% of her 213 million residents being under 19 years of age.^1^ A 10% prevalence^2^ suggests that over 10 million children and young people could be affected by mental health conditions in the country. Unfortunately, this large burden receives little attention and investment.^3^ Thus, most children and young people with mental health conditions do not have access to care, largely because of a severe shortage of trained child and adolescent mental health (CAMH) professionals. This results in a huge treatment gap for children and young people with mental health concerns in Nigeria.

The shortage of trained CAMH professionals has driven alternative service delivery models such as task sharing. This involves delegating tasks to existing or new cadres of health workers or lay persons trained to deliver CAMH interventions.^4^ However, the success of task sharing requires the availability of CAMH-trained professionals to develop policy and to provide leadership, advocacy, training and supervision for the non-CAMH professionals.^5^ A systematic review ^6^ found only one short (3 days) CAMH training programme from Nigeria and none from other West African countries. Thus, the purpose of this paper is to describe the implementation of a long-term postgraduate CAMH training programme in Nigeria.

## Location and setting

Nigeria is Africa's most populous country, a lower-middle-income country and Africa's largest economy. The country has about 300 psychiatrists^7^ and no integrated policy or plan for CAMH,^8^ although some CAMH programmes are found in policy documents. Most Nigerians live in rural areas, but the few CAMH resources are located in cities.

The CAMH training programme described here is based at the University of Ibadan, located in south-western Nigeria, the oldest university in Nigeria and a World Health Organization (WHO) Collaborating Centre in mental health. Ibadan is the third largest city in the country, with a population of 3.6 million.

## Programme description

### Background

The programme is an international effort that the MacArthur Foundation seed-funded for 5 years. It was developed and delivered through a partnership between the University of Ibadan, Harvard Medical School, Imperial College London, and Sangath (Goa, India).

The 18-month master's programme provides CAMH training and practical experience in the first year, followed by 6 months of field research work. The programme was planned and designed with the involvement of all local and international collaborators. An on-site joint faculty workshop was used to finalise the curriculum and learning outcomes. All collaborating centres contributed to on-site teaching in the first few years until growth of local academic staff led to less need for international teaching contributions. The Harvard Medical School and Imperial College London teaching contributions were by Nigerian child and adolescent psychiatrists based in the diaspora, but who had trained and worked in Nigeria. They were thus familiar with the local context. The local faculty was rich in diversity and included child and adolescent psychiatrists, general psychiatrists, experts in intellectual disability, general paediatrics, paediatric neurology, mental health nursing, psychology and counselling, family intervention, pharmacology, public mental health, maternal mental health, ethics, health promotion and education, communication and language, medical law and child safeguarding, epidemiology and statistics, qualitative and quantitative research methods, and creative writing.

Prospective students are required to have completed a first degree. Owing to the need to train a broad range of professionals in CAMH, the programme was designed to be both inter- and intra-professional. Interprofessional training was a particularly important aim of the programme. This is because the severe shortage of core mental health professionals in Nigeria and other low- and middle-income countries (LMICs) makes it imperative to expand CAMH capacity by training a wider group of healthcare providers. Also, interprofessional training allows different professional groups to learn from each other during the programme and it reduces unhelpful interprofessional boundaries.^9^ Interprofessional training is supported by the WHO^10^ and has been successfully adopted by training programmes to improve CAMH capacity in other African countries, such as Uganda.^11^ Thus, for this programme, in addition to students with pre-existing mental health training (e.g. psychiatrists and psychologists), the training is open to other professionals who have experience of working with children and adolescents, such as those with background in paediatrics, nursing, education and social work.

### Curriculum

The curriculum was designed so that graduates could be expected to run CAMH services, provide leadership, training and supervision, and conduct CAMH research. The inclusion of a leadership module was in response to a *Lancet* report^12^ that identified lack of expertise in policy and system implementation as a hindrance to the development of CAMH in LMICs. The research module is in recognition of the need for graduates to be able to generate and interpret data and to increase contextually relevant research output, thereby reducing the ‘10/90’ research gap in LMICs.^13^ Students with an existing prescribing licence (e.g. doctors) participate in a psychopharmacology module to gain additional expertise in CAMH-related prescribing.

### Teaching

The class sizes support small group teaching strategies. Thus, the classroom elements of the course involve interactive lectures, workshops and seminars. Live synchronous teaching is supported with asynchronous resources, including videos, reading materials such as the free International Association for Child and Adolescent Psychiatry and Allied Professions (IACAPAP) e-textbook^14^ and WHO Mental Health Gap Action Programme (mhGAP) resources. Clinical training is delivered through supervised participation in CAMH, paediatric neurology, liaison psychiatry and intellectual disability clinics, attachments to community and residential special education centres, and visits to the residential component of the juvenile justice system and schools. All students carry out a course of supervised psychological intervention with a patient. The leadership training includes creative writing and regular practice of public speaking and presentations. Each student conducts a supervised primary research project in the areas highlighted as important for LMICs, including epidemiology, health systems and locally contextualised interventions.^13,15^

### Assessment

All the students participate in regular written formative assessments designed primarily to help them and the lecturers to track progress. However, to ensure good engagement with these formative assessments, students are made aware that the results contribute 30% towards the final grade. Each module has a final summative written examination, which contributes 70% of the final grade. All the students gain clinical CAMH competencies, which are assessed regularly in clinical settings and signed off in their logbooks. The research module's assessment requires a public presentation before an audience of other students, faculty and two external examiners, and a *viva voce* with two external examiners. Students who pass all modules are awarded a master's degree in CAMH. A postgraduate diploma award was subsequently introduced for students wishing to pursue all but the research module.

The course evaluation is bidirectional in that students also provide anonymous online feedback on the faculty's contributions. The programme director uses the feedback to address any areas of improvement with respective faculty members. The faculty with the most favourable feedback receives a ‘Faculty of the Year Award.’

### Output/outcome

The programme is in its 10th year at the time of writing and it has achieved a wide range of successful outcomes. In total, the programme has trained 166 CAMH professionals (54 males and 112 females) from 14 African countries (Nigeria, Cameroun, Eritrea, Gambia, Ghana, Kenya, Liberia, Malawi, Rwanda, Sierra Leone, Somalia, Tanzania, Zambia and Zimbabwe). The programme's goal of interprofessional training has been achieved, as the graduates include psychiatrists, paediatricians, medical officers, nurses, pharmacists, psychologists, counsellors, physiotherapists, occupational therapists, communication and language specialists, health education specialists and specialist teachers. Four graduates are now faculty and another three are administrators on the programme – thus contributing to its long-term sustainability. Several graduates are currently in international CAMH leadership positions (vice presidency of IACAPAP and presidency and executives of the African Association for Child and Adolescent Mental Health). One graduate is completing a PhD at the University of Cambridge. The students’ research projects have produced 23 peer-reviewed publications – including pioneering work on locally adapted psychological interventions.^15,16^

## Lessons learned, implications for other LMICs and challenges

The programme is underpinned by a ‘train-the-trainer’ approach to enhance its sustainability. This is an important goal, given that only 2 of 37 previous mental health training programmes in Africa had a train-the-trainer ethos.^6^ This approach meant that by the end of the seed funding, the programme had trained and mentored enough graduates to assume faculty and administrative positions, which allowed substantial reduction in the need for international faculty. Currently, only one international faculty is directly involved and their role is mainly to mentor ongoing development of local faculty. This role is now being achieved remotely, leading to further substantial cost reductions.

It is relevant that the two international faculty from Harvard Medical School and Imperial College London are also Nigerians. Thus, they were already familiar with the local context – so did not need expensive and time-consuming travel arrangements or lengthy orientations and acclimatisation. The benefit has been bidirectional as the international faculty deepened their cultural sensitivity, which is relevant to their work in the USA and the UK.

Low cost is critical for the programme. Thus, the teaching uses free reference resources such as the IACAPAP textbook, WHO mhGAP and publicly available free-access publications and teaching resources.

The programme has not been without challenges and potential barriers. As expected, the COVID-19 pandemic disrupted the programme, but the successful adoption of some remote delivery became a positive benefit which has been maintained post-pandemic. The current worldwide economic downturn has affected the number of self-funding students. The programme is delivered in English, so it is currently accessible only to students who are fluent in the language. Although interprofessional training is a strength of the programme, flexing the curriculum and delivery strategies for students with a wide range of professional backgrounds can also be a challenge. This requires several strategies, including faculty with a wide range of expertise, careful and active formative assessments for early and prompt identification of areas for further flexibility and adaptations, making capital out of the diversity of experiences in the group and facilitating interprofessional peer-to-peer support. Stability in institutional industrial relationships and in the wider sociopolitical context is also crucial for successful implementation.

Although there are other examples of interprofessional CAMH training programmes in LMICs^17^ and some specialist postgraduate CAMH training schemes specific for psychiatrists, we are unaware of other sustained, long-term, clinically oriented, interprofessional master's degree level CAMH training programmes in LMICs. Thus, the main goal of this paper is to share our learning as a means of encouraging others to consider starting similar long-term CAMH training programmes in other LMICs.

## Conclusions

Over the past 10 years this programme has been successful in training a wide spectrum of professionals from a wide range of African countries to gain CAMH experience and expertise. Many of the graduates are running clinical CAMH services in their countries, mostly pioneered by them. They are also conducting CAMH training, including as faculty on the programme, and some are in international CAMH leadership roles. The key success elements of the programme, which could be replicable in other LMICs, include international collaboration (particularly involving international faculty who are already familiar with the local context), adopting a train-the-trainer approach and using a curriculum that covers clinical aspects of CAMH while also developing leadership and research skills. The use of existing free training resources and access to seed funding also contributed to the success. Overall, the programme has demonstrated a replicable model for building CAMH capacity in LMICs.

## Data Availability

Data availability is not applicable to this article as no new data were created or analysed in this study.
